# Enhanced Microwave Absorption and Surface Wave Attenuation Properties of Co_0.5_Ni_0.5_Fe_2_O_4_ Fibers/Reduced Graphene Oxide Composites

**DOI:** 10.3390/ma11040508

**Published:** 2018-03-28

**Authors:** Yinrui Li, Dongmeng Li, Jing Yang, Hui Luo, Fu Chen, Xian Wang, Rongzhou Gong

**Affiliations:** School of Optical and Electronic Information, Huazhong University of Science and Technology, Wuhan 430074, China; li_yinrui@163.com (Y.L.); eileen_lee@hust.edu.cn (D.L.); quark@hust.edu.cn (J.Y.); zs101141@163.com (F.C.); wangx@hust.edu.cn (X.W.); rzhgong@hust.edu.cn (R.G.)

**Keywords:** Co_0.5_Ni_0.5_Fe_2_O_4_ fiber, RGO, microwave absorption properties, surface wave attenuation

## Abstract

Co_0.5_Ni_0.5_Fe_2_O_4_ fibers with a diameter of about 270 nm and a length of about 10 μm were synthesized by a microemulsion-mediated solvothermal method with subsequent heat treatment. The Co_0.5_Ni_0.5_Fe_2_O_4_ fibers/reduced graphene oxide (RGO) composite was prepared by a facile in-situ chemical reduction method. The crystalline structures and morphologies were investigated based on X-ray diffraction patterns and scanning electron microscopy. Magnetization measurements were carried out using a vibrating sample magnetometer at room temperature. Co_0.5_Ni_0.5_Fe_2_O_4_ fibers/RGO composites achieve both a wider and stronger absorption and an adjustable surface wave attenuation compared with Co_0.5_Ni_0.5_Fe_2_O_4_ fibers, indicating the potential for application as advanced microwave absorbers.

## 1. Introduction

Microwave absorbing materials with the properties of thinness, lightweight, broadband and strong absorption have received increasing attention due to their pragmatic and effective functions for reducing electromagnetic interference (EMI) pollution and defense stealth technology. The specular reflection, cavity or ducting scattering, and angular scattering as the major potential scattering source of stealth objects can be sufficiently suppressed by shaping of the objects and the use of radar absorbing materials (RAM) [1,2,3,4,5,6,7]. As far as we know, there is almost no work discussing reflection loss and surface wave attenuation together [8]. Therefore, there is an urgent need to prepare a new generation of absorbers to realize low reflection loss and strong surface wave attenuation together.

In recent years, extensive attention has been aroused regarding magnetic shape-controlled materials due to their technological applications such as medical diagnostics, high-density magnetic recording media, optical and mechanic devices, catalysts, advanced magnetic materials, and Ferro-fluids [9,10,11,12,13,14]. It is also reported that peculiar morphologies of the materials are also beneficial to their microwave absorption properties [15,16]. Besides the typical ferromagnetic materials such as iron, cobalt and nickel, spinel type ferrites also have been considered as one of the most promising microwave absorbing materials due to their low cost and high abundance [17,18,19,20]. But the high density and low surface area limit their applications as absorbers [21]. Compared with traditional ferrite materials, carbon-based composite materials have advantages, such as low density and high surface area, which can promote the microwave absorption ability [22,23,24,25,26,27,28]. Reduced graphene oxide with internal residual defects and groups not only improves the impedance match characteristic but also introduces polarization relaxation and electronic dipole relaxation, which are beneficial for microwave penetration and absorption [29]. The microwave absorption properties of Co_0.5_Ni_0.5_Fe_2_O_4_ fibers and reduced graphene oxide composites have not been reported.

In this study, we report the microwave absorption and surface wave attenuation properties of Co_0.5_Ni_0.5_Fe_2_O_4_ fibers/RGO composites. The results show that Co_0.5_Ni_0.5_Fe_2_O_4_ fibers and the reduced graphene oxide worked synergistically as the magnetic loss absorber and dielectric loss absorber, respectively, and enhanced the microwave absorption properties. This work evaluates reflection loss and surface wave attenuation of one absorber simultaneously. More interestingly, Co_0.5_Ni_0.5_Fe_2_O_4_ fibers/RGO composites show great surface wave attenuation properties with adjustable attenuation frequency band, thus making it an advanced absorber.

## 2. Experimental Section

In a typical preparation, graphene oxide (GO) was prepared from natural graphite by using a modified Hummers method [30]. The precursor of Co_0.5_Ni_0.5_Fe_2_O_4_ fibers were synthesized in a microemulsion system. In the typical procedure, CTAB (1.6 g) was dissolved in a mixture of 120 mL cyclohexane and 4 mL n-pentanol and stirred for 30 min. Then, a mixed aqueous solution (6 mL) containing H_2_C_2_O_4_·H_2_O (1.21 g) was added to the mixture and stirred for another 1 h. Finally, 2 mL aqueous solution containing CoSO_4_·7H_2_O (0.028 g), NiSO_4_·6H_2_O (0.026 g) and FeSO_4_·7H_2_O (0.111g) was added to the microemulsion drop by drop. After substantial stirring, the microemulsion was transferred into a stainless Teflon-lined autoclave and heat at 80 °C for 6 h, then cooled to room temperature naturally. The solid product was washed with propanone and ethanol several times and then dried. The Co_0.5_Ni_0.5_Fe_2_O_4_ fibers were obtained by heat treatment of the precursors of Co_0.5_Ni_0.5_Fe_2_O_4_ fibers at 700 °C for 3 h. The Co_0.5_Ni_0.5_Fe_2_O_4_ fibers/reduced graphene oxide composite was synthesized in the solution by stirring suspension of Co_0.5_Ni_0.5_Fe_2_O_4_ fibers (100 mg) and graphene oxide (GO) powders (100 mg) in a mixture of 100 mL water and 5 mL aqueous hydrazine (N_2_H_4_·H_2_O, 80% concentration) for 2 h at 90 °C. After being centrifuged and dried, the Co_0.5_Ni_0.5_Fe_2_O_4_ fiber/RGO composites were obtained.

The crystalline structures of the as-synthesized samples were studied by X-ray powder diffraction (XRD, XRD-7000, SHIMADZU, Kyoto, Japan) using Cu Kα radiation (λ = 0.154187 Å), with the scanning rate of 0.02° S^−1^ in the 2θ range of 5°–70°. Scanning Electron Microscopy (SEM, Nova NanoSEM 450, FEI, Shanghai, China) was used to characterize the morphology of the samples. Magnetic properties were studied using a Lakeshore 7404 vibrating sample magnetometer with an applied magnetic field of ±15 kOe. Toroidal-shaped samples (inner diameter 3.04 mm, outer diameter 7 mm) with the weight ratio of samples to paraffin 3:7 were prepared to fit well the coaxial sample holder for microwave measurements. The complex permeability and permittivity of the test samples were examined over the frequency range of 300 MHz–18 GHz by reflection and transmission method carried out by an Agilent E5071C vector network analyzer.

## 3. Results and Discussion

**Figure 1:**
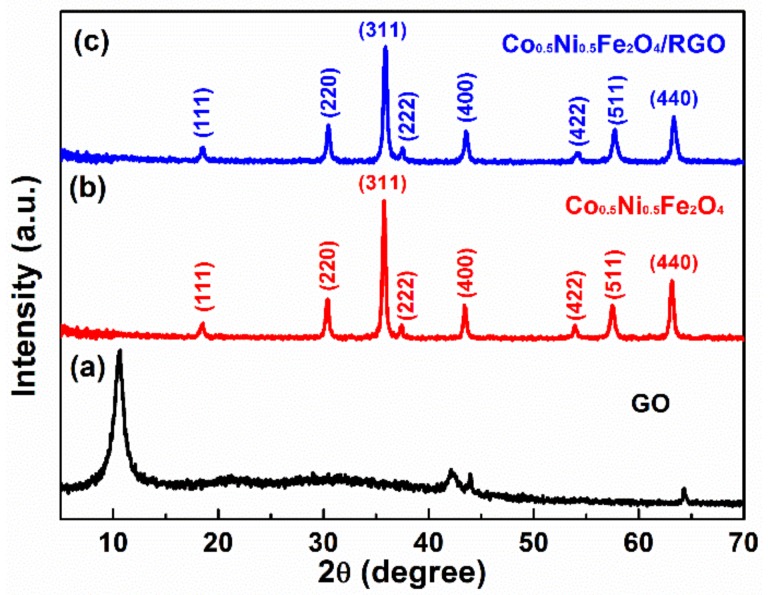
XRD patterns of the samples: (**a**) GO; (**b**) Co_0.5_Ni_0.5_Fe_2_O_4_ fibers; (**c**) Co_0.5_Ni_0.5_Fe_2_O_4_ fibers/RGO composites.

Figure 1 shows the typical X-ray diffraction (XRD) patterns of GO, Co_0.5_Ni_0.5_Fe_2_O_4_ fibers and Co_0.5_Ni_0.5_Fe_2_O_4_ fibers/RGO composites. Figure 1a shows the XRD pattern of GO. All the main peak values in the pattern in Figure 1b could be an index of the cubic crystalline phase of NiFe_2_O_4_ (JCPDS card 03-0875) or CoFe_2_O_4_ (JCPDS card 01-1121) with little deviation. The deviation is mainly caused by the (511) peak value 57.40°, of which the NiFe_2_O_4_ is 57.56°, CoFe_2_O_4_ is 57.17°. This is mainly due to the variety of the lattice constants. As the lattice constants increase, the diffraction peaks shift left [31,32]. The last diffraction pattern in Figure 1c shows the Co_0.5_Ni_0.5_Fe_2_O_4_ fibers/RGO composites. There is no diffraction peak around 2θ = 26° in the Co_0.5_Ni_0.5_Fe_2_O_4_ fibers/RGO composites, indicating that Co_0.5_Ni_0.5_Fe_2_O_4_ fibers are efficiently assembled on the surface of RGO, suppressing the stacking of graphene layers and destroying the (002) interplanar periodic structure [33,34,35].

**Figure 2:**
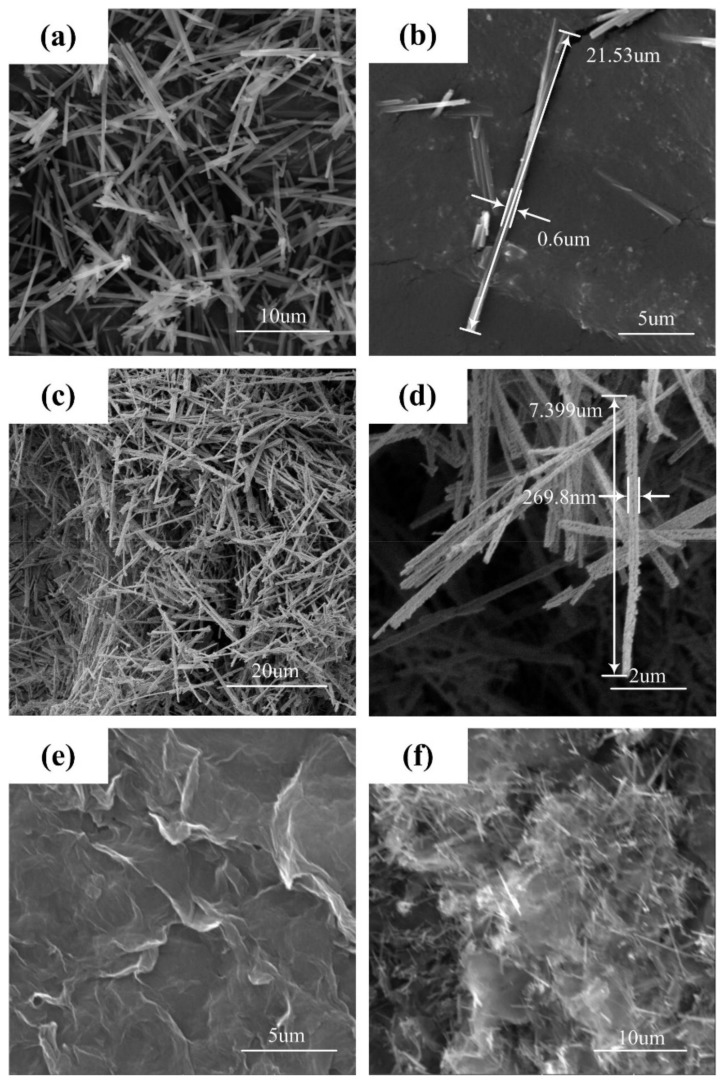
SEM images of the as-prepared powders: (**a**,**b**) Co/Ni/Fe composite oxalate fibers; (**c**,**d**) Co_0.5_Ni_0.5_Fe_2_O_4_ fibers; (**e**) GO; (**f**) Co_0.5_Ni_0.5_Fe_2_O_4_ fibers/RGO composites.

The as-prepared Co/Ni/Fe composites oxalate fibers have a mono-disperse and uniform fiber-like morphology as shown in Figure 2a. Figure 2b illustrates the precursor fibers have an average diameter of 600 nm and length of about 10 μm. From the typical SEM image (Figure 2c), the morphology of the Co_0.5_Ni_0.5_Fe_2_O_4_ fibers shows little change after calcination. The diameter of the Co_0.5_Ni_0.5_Fe_2_O_4_ fibers decreases to about 200 nm with little change in length, and the surface of the fibers become a little rough, as shown in Figure 2d. The Co_0.5_Ni_0.5_Fe_2_O_4_ fiber precursor is initially amorphous, but upon heat treatment, the Co_0.5_Ni_0.5_Fe_2_O_4_ fibers nucleate internally and crystalline through not only “oriented attachment” but also directional coalescence, thus leading to the rough surface of the Co_0.5_Ni_0.5_Fe_2_O_4_ fibers [36,37]. Figure 2e shows the prefabricated GO with layered structure. The Co_0.5_Ni_0.5_Fe_2_O_4_ fibers are dispersed homogeneously and embedded inside as well as on the surface of the RGO, and the composites form large stratiform particles, as shown in Figure 2f.

**Figure 3:**
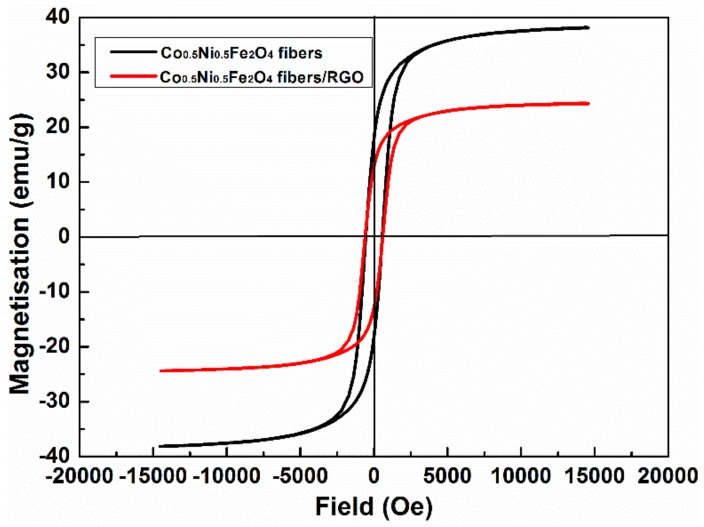
Magnetic hysteresis curves for Co_0.5_Ni_0.5_Fe_2_O_4_ fibers and Co_0.5_Ni_0.5_Fe_2_O_4_ fibers/RGO composites measured at room temperature.

The magnetic hysteresis curves for Co_0.5_Ni_0.5_Fe_2_O_4_ fibers and Co_0.5_Ni_0.5_Fe_2_O_4_ fibers/RGO composites were measured at room temperature, as shown in Figure 3. The saturation magnetization (Ms) of Co_0.5_Ni_0.5_Fe_2_O_4_ fibers and Co_0.5_Ni_0.5_Fe_2_O_4_ fibers/RGO composites are 38.17 emu/g and 24.39 emu/g, respectively. The measured saturation magnetizations of Co_0.5_Ni_0.5_Fe_2_O_4_ fibers/RGO composites are lower than those of Co_0.5_Ni_0.5_Fe_2_O_4_ fibers because the RGO is diamagnetic at room temperature [29]. The coercivity of the Co_0.5_Ni_0.5_Fe_2_O_4_ fibers and Co_0.5_Ni_0.5_Fe_2_O_4_ fibers/RGO is almost the same.

**Figure 4:**
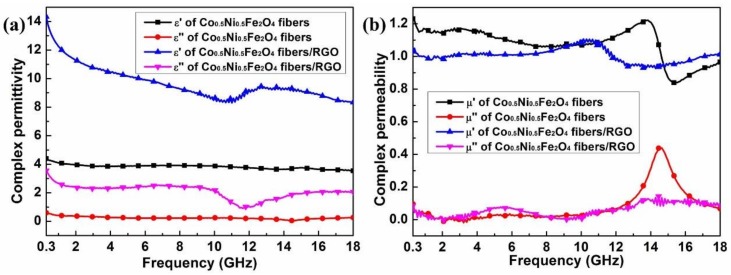
Frequency dependence of the relative complex electromagnetic parameters spectra: (**a**) Complex permittivity; (**b**) Complex permeability.

The microwave absorbing properties of the materials are dominated by the magnetic and dielectric losses [17]. Figure 4 shows the measured complex permittivity and complex permeability in the frequency range of 0.3–18 GHz. Generally speaking, the real parts of complex permittivity and permeability symbolize the storage ability of electromagnetic energy, and the imaginary parts represent the electromagnetic energy loss ability [38]. According to the free electron theory, $\varepsilon \prime \prime \approx 1/2\pi \rho f\varepsilon_{0}$, where ρ, f and $\varepsilon_{0}$ are the resistivity, the frequency and the dielectric constant of free space, respectively. It is found that both the real and imaginary parts of complex permittivity of the Co_0.5_Ni_0.5_Fe_2_O_4_ fibers/RGO are obviously higher than Co_0.5_Ni_0.5_Fe_2_O_4_ fibers because RGO possesses high conductive properties. The polarization loss can be divided into dipolar orientation polarization and interfacial polarization at microwave frequency [39]. The difference in dielectric constants and electrical conductivities among the paraffin, RGO and Co_0.5_Ni_0.5_Fe_2_O_4_ fibers is responsible for the generation of interfacial polarization. For all samples, the variation of both the real and imaginary parts of the complex permeability is small, indicating a certain amount of magnetic loss contributions. Apart from dielectric loss and magnetic loss, another important concept relating to microwave absorption is the impedance match characteristic: too high permittivity of absorber is harmful to the impedance match and results in strong reflection and weak absorption [40].

To evaluate the electromagnetic absorption performance of the samples, the reflection loss (RL) values were calculated based on the experimental data of the complex permittivity and permeability, and a singer layer of the composite sample was assumed to be attach on a metal plate. The reflection loss RL (dB) is calculated by the following equations: [41,42]

(1) $$RL=20\log\left|\left(Z_{in}-Z_{0}\right)/\left(Z_{in}+Z_{0}\right)\right|$$

(2) $$Z_{in}=Z_{0}\sqrt{\left(\mu_{r}/\varepsilon_{r}\right)}\tanh\left[j\left(2\pi fd/c\right)\sqrt{\mu_{r}\varepsilon_{r}}\right]$$

where $Z_{0}$ is the impedance of free space, $Z_{in}$ is the input impedance of the absorber, f is the frequency of the electromagnetic waves, c is the velocity of electromagnetic waves in free space, $\mu_{r}$ and $\varepsilon_{r}$ are, respectively, the relative complex permeability and permittivity, and d is the thickness of the absorber.

**Figure 5:**
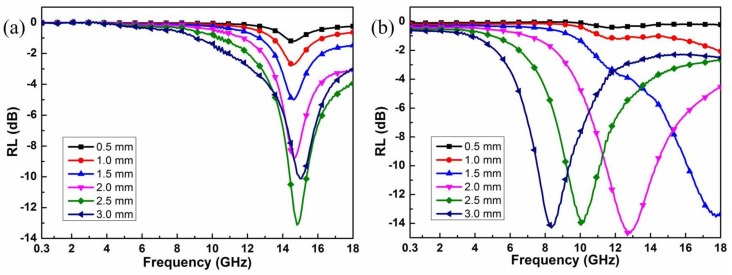
The calculated reflection loss: (**a**) Co_0.5_Ni_0.5_Fe_2_O_4_ fibers; (**b**) Co_0.5_Ni_0.5_Fe_2_O_4_ fibers/RGO.

The reflection loss (RL) of Co_0.5_Ni_0.5_Fe_2_O_4_ fibers and Co_0.5_Ni_0.5_Fe_2_O_4_ fibers/RGO with different thickness is shown in Figure 5. The thickness of the sample is one of the crucial parameters which affects the intensity and the position of the frequency at the reflection loss minimum [21]. This phenomenon has been successfully explained by the quarter-wavelength cancellation model in absorbers [43]. The maximum reflection loss reaches −13.1 dB at 14.8 GHz and the absorption frequency range under −10 dB (90% of EM wave absorption) is from 14.4 to 15.4 GHz for the Co_0.5_Ni_0.5_Fe_2_O_4_ fibers containing samples with a thickness of 2.5 mm. With the variety of the thickness, the reflection loss under −5 dB can be only tuned from 13.6 to 16.7 GHz. As shown in Figure 5b, the reflection loss of the sample containing Co_0.5_Ni_0.5_Fe_2_O_4_ fibers/RGO less than −10 dB was obtained in the 1.9 GHz (from 16.1 to 18 GHz) with the thickness of 1.5 mm. It is worth noting that the sample with a thickness of 2.0 mm shows a minimum reflection loss value of −14.7 dB at 12.9 GHz. The minimum reflection loss peak moves drastically towards the lower frequency region with increasing thickness of the sample. With the variety of thickness, the effective absorption band width (RL ≤ −10 dB) can be tuned from 7.5 to 18 GHz, indicating adjustable microwave absorption properties.

Apart from dielectric loss and magnetic loss, impedance matching characteristic is another important parameter for microwave absorption and is calculated by the following equation: [44]

(3) $$\left|Z_{in}/Z_{0}\right|={\left|\mu_{r}/\varepsilon_{r}\right|}^{1/2}\left|\tanh\left[j\left(2\pi fd/c\right)\sqrt{\mu_{r}\varepsilon_{r}}\right]\right|$$

**Figure 6:**
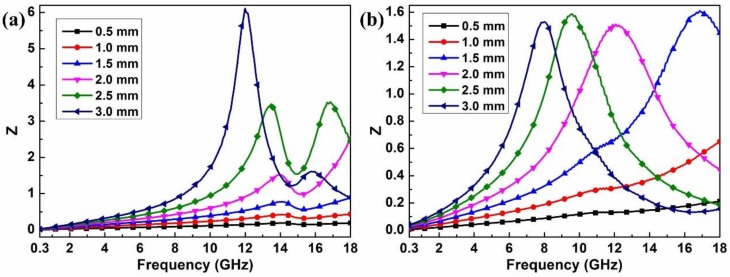
The modulus of normalized input impedance $\left(\left|Z_{in}/Z_{0}\right|\right)$: (**a**) Co_0.5_Ni_0.5_Fe_2_O_4_ fibers; (**b**) Co_0.5_Ni_0.5_Fe_2_O_4_ fibers/RGO.

The frequency dependence of $Z=\left|Z_{in}/Z_{0}\right|$ for the Co_0.5_Ni_0.5_Fe_2_O_4_ fibers and Co_0.5_Ni_0.5_Fe_2_O_4_ fibers/RGO composites was obtained, as presented in Figure 6. It is illustrated in Figure 6b that the minimum RL of Co_0.5_Ni_0.5_Fe_2_O_4_ fibers/RGO composites can be obtained and the corresponding *Z* is most close to 1 when the matching frequency is 12.9 GHz and the matching thickness is 2.0 mm. On the contrary, the impedance matching characteristic of Co_0.5_Ni_0.5_Fe_2_O_4_ fibers is poor compared with Co_0.5_Ni_0.5_Fe_2_O_4_ fibers/RGO composites, as shown in Figure 6a.

The enhanced absorption properties of Co_0.5_Ni_0.5_Fe_2_O_4_ fibers/RGO composites can be explained by the following facts. First, the multi-interfaces and triple junctions (RGO@Co_0.5_Ni_0.5_Fe_2_O_4_, RGO@paraffin, Co_0.5_Ni_0.5_Fe_2_O_4_@paraffin) are advantageous for electromagnetic attenuation due to the existing interfacial polarization [45]. Second, RGO provides a large receptive surface for individual dispersion of Co_0.5_Ni_0.5_Fe_2_O_4_ fibers and acts as an excellent substrate for the absorption of microwave, providing more active sites for reflection and scattering of electromagnetic waves, which makes both dielectric loss and magnetic loss work synergistically [46]. Third, the defects in the RGO can act as polarization and scatting centers introducing dielectric loss. Fourth, the impedance matching changed after the RGO was introduced, which can be seen in Figure 6.

Evaluating reflection loss and surface wave attenuation together is a wise choice, because non-specular reflection resulting from surface wave also contributes substantially to the radar cross section (RCS). A single layer of the composite sample is assumed to be attached to an infinite metal plate, and the surface wave attenuation constants β″ were calculated based on the classic dispersion equations: [47]

(4) $$D\left(k_{0},\beta \right)\equiv \sqrt{k_{0}^{2}\varepsilon_{r}\mu_{r}-\beta^{2}}\tan\left(d\sqrt{k_{0}^{2}\varepsilon_{r}\mu_{r}-\beta^{2}}\right)+j\varepsilon_{r}\sqrt{k_{0}^{2}-\beta^{2}}=0$$

where $k_{0}=\omega \sqrt{\varepsilon_{0}\mu_{0}}$ is the wave number of the free space, d is the thickness of the absorber. The quantity β is the longitudinal wave number, or the propagation constant, and β″, the imaginary part of β, is the surface wave attenuation constant.

**Figure 7:**
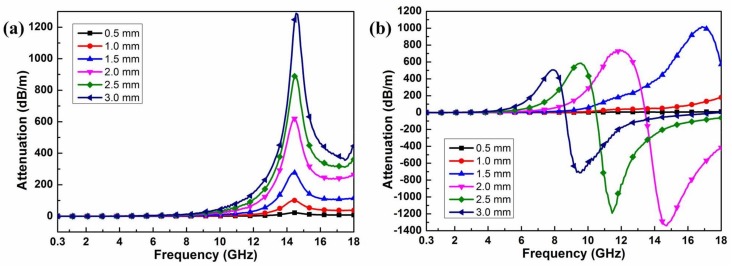
The calculated surface wave attenuation constants: (**a**) Co_0.5_Ni_0.5_Fe_2_O_4_ fibers; (**b**) Co_0.5_Ni_0.5_Fe_2_O_4_ fibers/RGO.

The surface wave attenuation constant (β″) of Co_0.5_Ni_0.5_Fe_2_O_4_ fibers and Co_0.5_Ni_0.5_Fe_2_O_4_ fibers/RGO with different thickness is shown in Figure 7. All the samples with the thickness of 0.5 mm show weak attenuation properties. There are nearly no attenuation abilities in the frequency range of 0.3–8 GHz for the Co_0.5_Ni_0.5_Fe_2_O_4_ fibers samples with different thickness, as shown in Figure 7a. The attenuation reaches a certain intensity in the frequency range of 12–18 GHz which can attenuate surface waves sufficiently. But after the attenuation constants reach the maximum value at about 14.3 GHz, then decrease sharply. As for the Co_0.5_Ni_0.5_Fe_2_O_4_ fibers/RGO samples, the attenuation constants reach their maximum value and then decrease with a further increase in frequency. The attenuation constants reach zero values and then become negative. Negative values of β″ result from non-physical solutions of the dispersion equation. They imply the amplification of the field, rather than energy absorption, and must be ignored. Physically, these negative values mean that surface waves cannot be excited and thus no propagation occurs along the layers. The frequency at which the quantity β″ vanishes can be considered as the upper cutoff frequency [48,49]. As the thickness of the sample increases, the upper cutoff frequency moves to the lower frequency, as shown in Figure 7b. Compared with Co_0.5_Ni_0.5_Fe_2_O_4_ fibers, the attenuation frequency band of the sample Co_0.5_Ni_0.5_Fe_2_O_4_ fibers/RGO can be adjusted and maintain a high attenuation constant value, showing good surface wave attenuation properties.

## 4. Conclusions

In summary, Co_0.5_Ni_0.5_Fe_2_O_4_ fibers have been prepared by a microemulsion-mediated solvothermal method and heat treatment. The composites of Co_0.5_Ni_0.5_Fe_2_O_4_ fiber/RGO were prepared by the in-situ chemical reduction method. The Co_0.5_Ni_0.5_Fe_2_O_4_ fibers retained the original fiber-like morphology throughout the whole calcination procedure. The microwave absorption properties of Co_0.5_Ni_0.5_Fe_2_O_4_ fibers/RGO composites are higher compared with the Co_0.5_Ni_0.5_Fe_2_O_4_ fiber. The enhanced microwave absorption properties are due to the magnetic loss absorber Co-Ni ferrite fibers and dielectric loss absorber RGO working synergistically. The Co_0.5_Ni_0.5_Fe_2_O_4_ fiber/RGO composites show great surface wave attenuation properties with adjustable frequency band. This work provides a facile method for preparation of Co_0.5_Ni_0.5_Fe_2_O_4_ fiber/RGO composites with great potential application as an advanced absorber for microwave absorption, especially in situations where surface wave attenuation is highly demanded.
